# Hybrid emergency rooms reduce the requirement of blood transfusion in patients with severe trauma

**DOI:** 10.1186/s13017-021-00377-w

**Published:** 2021-06-26

**Authors:** Hiroaki Watanabe, Ryo Matsumoto, Shunsuke Kuramoto, Tomohiro Muronoi, Kazuyuki Oka, Yoshihide Shimojo, Akihiko Kidani, Eiji Hira, Toshihiko Kawamura

**Affiliations:** 1 Department of Acute Care Surgery, Shimane University Faculty of Medicine, 89-1 Enya-cho, Izumo, Shimane 693-8501 Japan; 2 Advanced Trauma Center, Shimane University Hospital, 89-1 Enya-cho, Izumo, Shimane 693-8501 Japan; 3 Division of Medical Informatics, Shimane University Hospital, 89-1 Enya-cho, Izumo, Shimane 693-8501 Japan

**Keywords:** Hybrid emergency room, Hemostasis, Blood transfusion

## Abstract

**Background:**

A hybrid emergency room (ER) is defined as an emergency unit with four functions—performing resuscitation, computed tomography (CT), surgery, and angiography. However, the safety and efficacy of performing CT in a hybrid ER are unclear in primary surveys. Therefore, this study aimed to evaluate the safety and clinical effects of hybrid ERs.

**Methods:**

This retrospective observational study used data from the Shimane University Hospital Trauma Database from January 2016 to February 2019. Hospitalized patients with severe trauma and an injury severity score of ≥ 16 were divided into the non-hybrid ER group (n = 134) and the hybrid ER group (n = 145). The time from arrival to CT and interventions and the number of in-hospital survivors, preventable trauma deaths (PTD), and unexpected survivors (US) were assessed in both groups. Further, the amount of blood transfused was compared between the groups using propensity score matching.

**Results:**

The time from arrival to CT and interventions was significantly reduced in the hybrid ER group compared to that in the non-hybrid ER group (25 vs. 6 min; *p* < 0.0001 and 101 vs. 41 min; *p* = 0.0007, respectively). There was no significant difference in the rate of in-hospital survivors (96.9% vs. 96.3%; p = 0.770), PTD (0% vs. 0%), and US (9.0 vs. 6.2%; p = 0.497) between the groups. The amount of blood transfused was significantly lower in the hybrid ER group than in the non-hybrid ER group (whole blood 14 vs. 8, *p* = 0.004; red blood cell 6 vs. 2, *p* = 0.012; fresh frozen plasma 9 vs. 6, *p* = 0.021). This difference was maintained after propensity score matching (whole blood 28 [10–54] vs. 6 [4–16.5], *p* = 0.015; RBC 8 [2.75–26.5] vs. 2 [0–8.5], *p* = 0.020, 18 [5.5–27] vs. 6 [3.5–7.5], *p* = 0.057).

**Conclusions:**

The study results suggest that trauma treatment in a hybrid ER is as safe as conventional treatment performed in a non-hybrid ER. Further, hybrid ERs, which can reduce the time for trauma surveys and treatment, do not require patient transfer and can reduce the amount of blood transfused during resuscitation.

## Background

Hemostasis is an essential element that can save the lives of patients with severe trauma [1]. Primary surveys in Advanced Trauma Life Support (ATLS) show that a simple assessment, including a focused assessment with sonography for trauma (FAST) and radiography of the chest and pelvis, is recommended to achieve resuscitation in patients with severe trauma. Therefore, computed tomography (CT) imaging requiring patient transfer should not be performed for hemodynamically unstable trauma patients. However, with the progress in CT equipment, an increasing number of studies have indicated that whole-body CT is effective for trauma treatment [2–8]. Based on these studies, a hybrid emergency room (ER) was developed to achieve not only accurate diagnosis but also faster surgical and angiographic hemostasis. This emergency unit utilizes new concepts to perform immediate initial resuscitation in patients with life-threatening injuries and perform trauma whole-body CT to facilitate quick damage control surgery and transcatheter arterial embolization (TAE) using interventional radiology (IVR). However, the safety of CT in primary surveys is unknown.

Massive transfusion is required for trauma resuscitation, including damage control surgery [9]. Damage control resuscitation (DCR) is an important strategy aimed at minimizing the amount of blood loss until achievement of definitive hemostasis [9, 10]. In addition to performing DCR, achieving hemostasis in patients with massive bleeding and additional life-threatening injuries is also essential. If a system that enables faster hemostasis can be established, it may be possible to reduce the amount of blood required for transfusion. The hybrid ER can be considered a leading candidate for this type of system. However, its application has not been verified in practice.

We hypothesized that trauma treatment in a hybrid ER is as safe as conventional resuscitation performed in a non-hybrid ER and that the use of hybrid ER can reduce the number of blood transfusions required. In the present study, we evaluated the safety of using a hybrid ER system for primary trauma care and assessed whether the use of hybrid ER could reduce the amount of blood transfused in patients with severe trauma.

## Methods

### Study design and patient selection

**Fig. 1:**
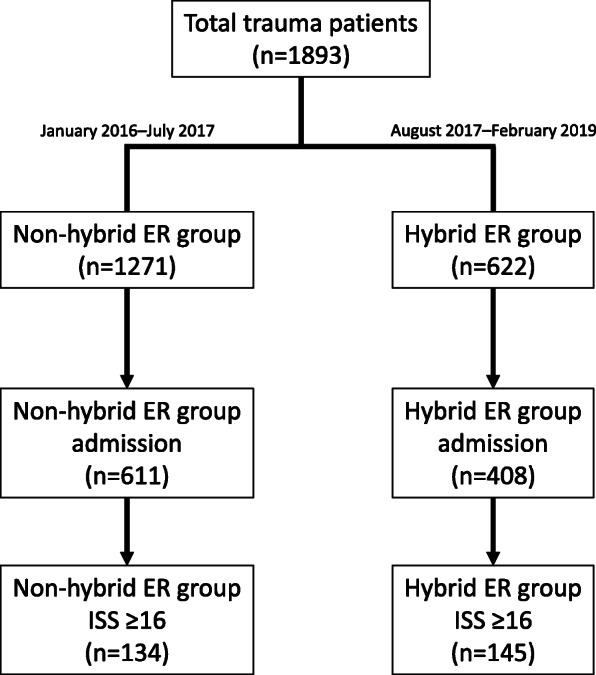
Flowchart of patient inclusion in the study. ER, emergency room; ISS, injury severity score

This retrospective observational study used data from the Shimane University Hospital Trauma Database from January 2016 to February 2019. This study was approved by the Shimane University Institutional Committee on Ethics (#4083). The Shimane University Hospital Trauma Database includes data collected from the medical records of patients who needed hospitalization or were transported by emergency ambulance or medical helicopter to Shimane University Hospital. Hospitalized patients with severe trauma (injury severity score [ISS] ≥ 16) were enrolled in this study. The patients were divided into the non-hybrid ER group (January 2016–July 2017) and the hybrid ER group (August 2017–February 2019) (Fig. 1).

### Management in non-hybrid ERs

The patients were transferred to a regular emergency room in our trauma center. The Japan Advanced Trauma Evaluation and Care (JATEC) guidelines [11], based on the ATLS guidelines, were applied to all the patients. FAST and radiography of the chest and pelvis for the assessment of circulation was performed in the primary survey. If patients needed resuscitation, it was immediately performed in the emergency room. Resuscitative thoracotomy was performed in the emergency room; however, emergency laparotomy and craniotomy were performed after patient transfer in the operating room. Whole-body CT was performed after the secondary surveys according to the JATEC guidelines. The CT room in the ER facility was located next to the standard ER. The operating room was on the third floor, and the interventional radiology department was very far from the operating room. If patients needed hemostasis using IVR, they were transferred to the angiographic room, where TAE was performed accordingly. Blood transfusion was immediately initiated if needed because transfusion products were permanently stored in our trauma center.

### Management in hybrid ERs

**Fig. 2:**
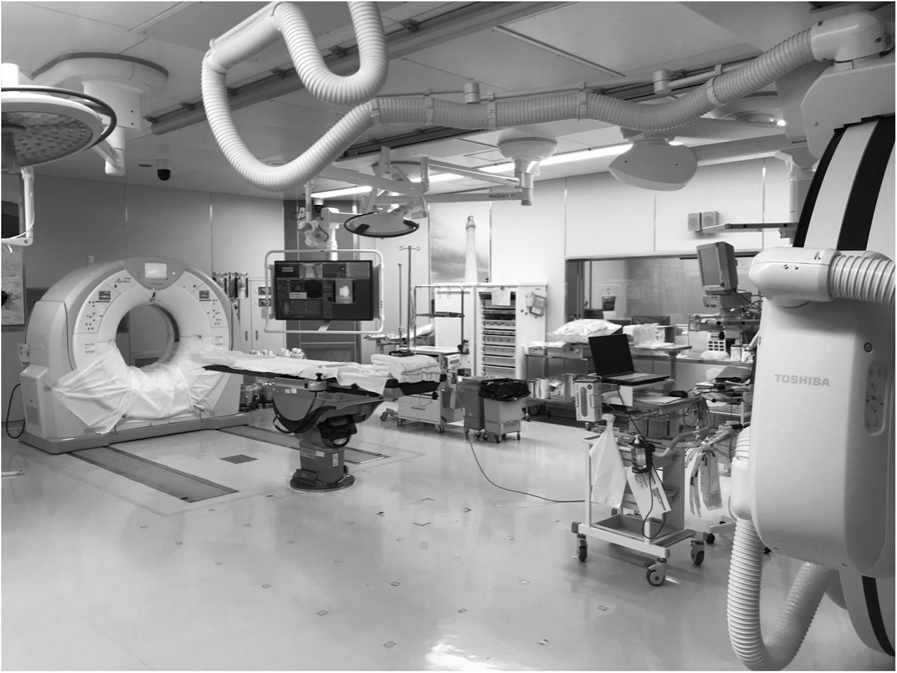
Hybrid emergency room used in this study

The Shimane Advanced Trauma Center of Shimane University Hospital installed a rotation-type hybrid ER system in August 2018 (Fig. 2) [12]. Our hybrid ER utilizes an operating table, not an angiographic table, and has an air-conditioned room (class 10,000) (12). Therefore, all surgeries, including craniotomy, can be performed in this hybrid ER without transferring the patients. Whole-body CT, damage control surgery, and TAE can be performed quickly after transferring patients to our trauma center. We created a clinical protocol for hybrid ER. The criteria for patients transferred to the hybrid ER were as follows: (1) high-energy trauma and (2) abnormalities in the airway, respiration, circulation, or consciousness according to pre-hospital information. The basic concept of the protocol conformed to the ABCDE approach as well as JATEC. After patient transfer to the hybrid ER, assessment of A (airway), B (breathing), and C (circulation) was performed. After assessment of airway and breathing, assessment of circulation was completed using whole-body CT instead of radiography of the chest and pelvis and FAST. If patients had airway or breathing abnormalities, resuscitation, including intubation, was accordingly prioritized. The team leader evaluated the following 10 life-threatening injuries: (1) intracranial bleeding requiring craniotomy, (2) pericardial effusion, (3) mediastinal hematoma and aortic injury, (4) massive hemothorax, (5) pneumothorax requiring a chest drain, (6) massive lung contusion with desaturation, (7) multiple rib fractures that could cause flail chests, (8) abdominal bleeding (organ injury or mesenteric injury), (9) retroperitoneal hematoma with pelvic fracture, and (10) massive retroperitoneal bleeding in zone I or II. We called the assessment of these cases using CT in the hybrid ER, “mFACT: modified focused assessment with CT for trauma.” If even one qualifying case was found, it was defined as “mFACT positive.” If mFACT was positive, resuscitation, including resuscitative surgery or IVR, was immediately performed. CNS dysfunction was assessed after whole-body CT. If it was determined that the patient might have a cardiac arrest soon (e.g., a systolic blood pressure of ≤ 60 mmHg), resuscitative thoracotomy was performed instead of whole-body CT. Damage control surgery, craniotomy, and TAE were performed immediately without patient transfer. The basic protocol for the primary survey in the hybrid ER followed the ABC“CT”DE approach.

### Data sources

Data were obtained from the Shimane University Hospital Trauma Database. Age, sex, mechanism of trauma, abbreviated injury scale (AIS) score, maximum AIS score, ISS, revised trauma score (RTS), probability of survival (Ps) calculated using the Trauma and Injury Severity Score (TRISS) [13, 14], cardiac pulmonary arrest on arrival (CPA-OA), blood transfusion, and procedure of resuscitative surgery or IVR, were compared between the groups. Resuscitative thoracotomy, laparotomy, pelvic external fixation, craniotomy, or TAE was performed for resuscitative surgery or IVR as an intervention. To estimate the safety of the hybrid ER, the prognosis of patients, in-hospital survival of patients, death in patients with Ps > 0.5 (excluding patients with severe head injuries of Glasgow coma scale [GCS] score ≤ 5 and those aged ≥ 80 years), survivors with Ps < 0.5, and complications were compared between the groups. Complications were assessed using the adapted Clavien–Dindo in trauma (ACDiT) [15]. Cases of CPA-OA were excluded from the evaluation of in-hospital survivors and complications. Finally, the amount of red blood cells (RBCs) and fresh frozen plasma (FFP) transfused in the emergency room was compared between the groups. A transfusion protocol for damage control resuscitation by the Shimane Advanced Trauma Center was used for this purpose. Blood transfusion was initiated based on the assessment of blood consumption score [16], but the final decision to initiate blood transfusion was made by the attending trauma surgeon. Complete blood count and coagulation tests were performed every 30 min, and the targeted goals for transfusion were a hemoglobin level of 7–9 g/dL and a fibrinogen level of > 150 mg/dL.

### Statistical analysis

Statistical analysis was performed using JMP Pro 14.2.0 (SAS Institute Inc., Tokyo, Japan). Differences in baseline characteristics between the groups were analyzed using the Wilcoxon test for continuous variables and the chi-square test and Fisher’s exact test for categorical variables. Differences in time from arrival to CT, time from arrival to interventions, and amount of blood transfused between the groups were analyzed using the Wilcoxon test. All results were assessed at a level of significance of *p* = 0.05. Propensity score matching analysis was used to match patients in the hybrid ER with non-hybrid ER groups in a 1:1 ratio to the nearest available matching based on the caliper width of 0.20. The covariates for propensity score matching included age, sex, mechanism of trauma, maximum AIS score, ISS, RTS, TRISS Ps, CPA-OA, with or without intervention and blood transfusion.

## Results

A total of 1893 patients with trauma were identified in the Shimane University Hospital Trauma Database from January 2016 to February 2019 (Fig. 1). Among the 1271 patients treated in the non-hybrid ER, 134 patients with ISS ≥ 16 required hospitalization. In contrast, among the 622 patients treated in the hybrid ER, 145 patients with ISS ≥ 16 required hospitalization. The median age of all patients was 69 years (interquartile range [IQR], 52–80 years), similar to that of trauma patients in the trauma registry database in Japan. Table 1 shows a comparison of patient baseline characteristics between the study groups. There was no significant difference in the baseline characteristics between the non-hybrid ER and hybrid ER groups. In addition, there was no significant difference in the number of patients requiring interventions, including surgery or IVR, (24 vs. 39, *p* = 0.086) or blood transfusions (26 vs. 24, *p* = 0.640) between the groups. Although the number of CPA-OA in the hybrid ER group was slightly higher than that in the non-hybrid ER group (4 vs. 11, *p* = 0.113), there was no significant difference between the groups.

**Table 1:** Baseline characteristics of patients

	Non-hybrid ER (n = 134)	Hybrid ER (n = 145)	*p* value
Age in years (median)	68 (55–79)	70 (52–81)	0.531
Male	93 (69.4%)	100 (68.9%)	1.000
Brunt	133 (99.3%)	143 (98.6%)	1.000
AIS score ≥ 3			
AIS (head)	69 (51.5%)	87 (60.0%)	0.106
AIS (face)	3 (2.2%)	4 (2.8%)	0.598
AIS (chest)	85 (63.4%)	91 (62.8%)	0.229
AIS (abdomen)	24 (17.9%)	20 (13.8%)	0.200
AIS (extremity)	31 (23.1%)	34 (23.4%)	0.827
AIS (External)	1 (0.7%)	0 (0%)	0.170
Maximum AIS score	4 (4–4)	4 (4–5)	0.124
ISS (median)	22 (18–33)	26 (18–35)	0.254
RTS	7.24 (range, 0–7.84)	6.76 (range 0–7.84)	0.156
TRISS Ps	0.798 (0.773–0.940)	0.769 (0.758–0.939)	0.303
CPA-OA	4 (3.0%)	11 (7.6%)	0.113
Intervention	24 (17.9%)	39 (26.9%)	0.086
Blood transfusion	26 (19.4%)	24 (16.6%)	0.640

*ER* emergency room, *AIS* abbreviated injury scale, *ISS* injury severity score, *RTS* revised trauma score, *TRISS* Trauma and Injury Severity Score, *Ps* probability of survival, *CPA-OA* cardiac pulmonary arrest on arrival

### Time from arrival to CT and interventions, including surgery and IVR

To determine the efficacy of the hybrid ER in reducing the trauma care time, the time from arrival to CT and interventions, including surgery and IVR, was compared between the non-hybrid ER and hybrid ER groups (Table 2). The time from arrival to CT was significantly reduced in the hybrid ER group compared to that in the non-hybrid ER group (median [IQR]: 25 [17–35.5] vs. 6 [4–8] min, *p* < 0.0001). Furthermore, the time from arrival to interventions (hemostatic procedures including surgery and TAE) was also significantly reduced in the hybrid ER group compared to that in the non-hybrid ER group (median [IQR] 101 [43.8–152.5] vs. 41 [20–72] min, *p* = 0.0007).

**Table 2:** Time from arrival to CT and interventions

	Non-hybrid ER (n = 134)	Hybrid ER (n = 145)	p value
CT scan (min)	25 (17–35.5)	6 (4–8)	< 0.0001
Interventions (min)	101 (43.8–152.5)	41 (20–72)	0.0007

*ER* emergency room, *CT* computed tomography

### Prognosis of patients with severe trauma

To determine the safety of a novel trauma workflow using a hybrid ER, the number of in-hospital survivors, complications, death in patients with Ps > 0.5, and survivors with Ps < 0.5 were compared between the groups (Table 3). There was no significant difference in the number of in-hospital survivors between the two groups (126 [96.9%] vs. 129 [96.3%], *p* = 0.770). Further, there was no significant difference in the incidence of complications between the groups (Table 3). There was no death in patients with Ps > 0.5, except in those with severe brain injuries and a GCS score of ≤ 5 and those aged ≥ 80 years in both groups. Moreover, there was no significant difference in the number of survivors with Ps < 0.5 between both groups (12 vs. 9; *p* = 0.497) (Table 4).

**Table 3:** In-hospital survivors and complications

	Non-hybrid ER (n = 130)	Hybrid ER (n = 134)	*p* value
In-hospital survivors	126 (96.9%)	129 (96.3%)	0.770
Complications (ACDiT)			
0	109 (83.8%)	116 (86.6%)	0.880
I	1 (0.8%)	0	0.480
II	15 (11.5%)	11 (8.2%)	0.312
III-a	0	0	
III-b	1 (0.8%)	2 (1.5%)	1.000
IV-a	1 (0.8%)	0	0.480
IV-b	0	0	
V-a	0	0	
V-b	3 (2.3%)	5 (3.7%)	0.724

*ER* emergency room, *ACDiT* adapted Clavien–Dindo in trauma

**Table 4:** Prognosis of both groups (preventable death and unexpected survivors)

	Non-hybrid ER (n = 134)	Hybrid ER (n = 145)	*p* value
Death in patients with Ps > 0.5	0	0	
Survivors with Ps < 0.5	12 (9.0%)	9 (6.2%)	0.497

*ER* emergency room, *Ps* probability of survival

### Amount of blood transfusion

**Fig. 3:**
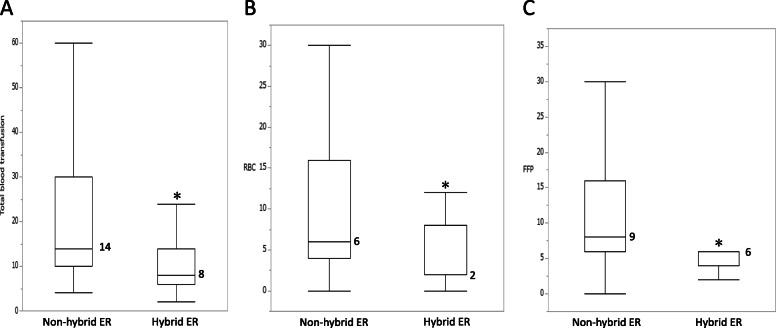
The amount of blood transfused in both groups. **A** Whole blood transfusion. **B** Red blood cell (RBC). **C** Fresh frozen plasma (FFP). *P < 0.05 for the comparison between the non-hybrid ER and hybrid ER groups

To assess the effect of hybrid ERs on trauma resuscitation, the amount of blood (whole blood, RBCs, and FFP) transfused from arrival to the end of the resuscitative interventions was assessed in both groups (Fig. 3). The total dose of RBCs was significantly lower in the hybrid ER group than in the non-hybrid ER group (6 [4–16] vs. 2 [2–8], *p* = 0.012). In point estimation, the non-hybrid ER group had higher RBC levels (9.81, 95% confidence interval [CI] 7.19–12.44) than the hybrid ER group (4.42, 95% CI 1.63–7.20). Furthermore, the amount of FFP transfused was significantly lower in the hybrid ER group than in the non-hybrid ER group (9 [6–17] vs. 6 [4–6], *p* = 0.021). In point estimation, the non-hybrid ER group had higher FFP levels (11.74, 95% CI 8.30–15.18) than the hybrid ER group (7.00, 95% CI 3.35–10.65). The total amount of the whole blood transfused, including RBCs and FFP, was significantly lower in the hybrid ER group than in the non-hybrid ER group (14 [10–30] vs. 8 [6–14], *p* = 0.004).

### Amount of blood transfusion after propensity score matching

**Fig. 4:**
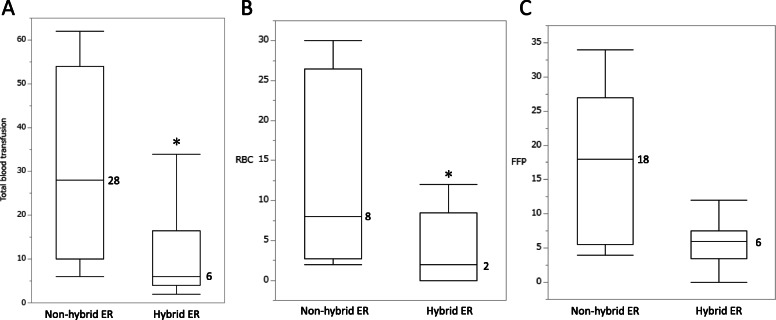
The amount of blood transfused in both groups after propensity score matching. **A** Whole blood transfusion. **B** Red blood cell (RBC). **C** Fresh frozen plasma (FFP). *P < 0.05 for the comparison between the non-hybrid ER and hybrid ER groups

Propensity score matching was performed to evaluate the effect of hybrid ERs on trauma resuscitation more accurately. The results are listed in Table 5. Each group consisted of 103 patients whose characteristics were balanced after propensity score matching. There were significant differences in the listed variables between the non-hybrid ER and hybrid ER groups (Table 5). The amount of blood (total blood, RBCs, and FFP) transfused was assessed in both groups after propensity score matching (Fig. 4). The total amount of whole blood and RBCs transfused were significantly lower in the hybrid ER group than in the non-hybrid ER group (whole blood 28 [10–54] vs. 6 [4–16.5], *p* = 0.015; RBC 8 [2.75–26.5] vs. 2 [0–8.5], *p* = 0.020). The amount of FFP transfused was lower in the hybrid ER group than in the non-hybrid ER group (18 [5.5–27] vs. 6 [3.5–7.5], *p* = 0.057). Our results indicated that the use of a hybrid ER, which shortened the time between arrival and interventions, including surgery, reduced the requirement for blood transfusion for resuscitation. There was no significant difference in mortality between the groups after propensity score matching (5 [4.9%] vs. 6[5.8%], *p* = 1.000).

**Table 5:** Baseline characteristics of patients after propensity score matching

	Non-hybrid ER (n = 103)	Hybrid ER (n = 103)	*p* value
Age in years (median)	70 (60–81)	69 (51–80)	0.511
Male	70 (68.0%)	70 (68.0%)	1.000
Brunt	102 (99.0%)	103 (100.0%)	1.000
Maximum AIS score	4 (4–4)	4 (4–4)	0.712
ISS (median)	21 (18–29)	24 (17–33)	0.465
RTS	7.84 (range, 0–7.84)	7.84 (range, 0–7.84)	0.588
TRISS Ps	0.916 (0.838–0.939)	0.911 (0.771–0.943)	0.654
CPA-OA	3 (2.9%)	3 (2.9%)	1.000
Intervention	15 (14.6%)	18 (17.5%)	0.705
Blood transfusion	9 (8.7%)	14 (13.6%)	0.377

*ER* emergency room, *AIS* abbreviated injury scale, *ISS* injury severity score, *RTS* revised trauma score, *TRISS* trauma and injury severity score, *Ps* probability of survival, *CPA-OA* cardiac pulmonary arrest on arrival

## Discussion

In the present study, we demonstrated two major findings—(1) trauma treatment in hybrid ERs did not increase the risk of mortality in patients with severe trauma compared with conventional treatment in non-hybrid ERs and (2) trauma resuscitation in a hybrid ER reduced the amount of blood transfusion required for resuscitation.

The hybrid ER system is defined as an integrated system that enables four functions to be performed in the same room without patient transfer—resuscitation, CT, surgery, and IVR [12, 17]. Trauma care using a hybrid ER has been associated with improved patient survival after severe trauma [18]. Furthermore, Kinoshita et al. reported that installation of a hybrid ER may significantly improve mortality in patients with severe trauma because of the capability of immediate diagnosis by CT and rapid control of massive bleeding without the need for patient transfer [19]. Further, recent studies have reported the efficacy of hybrid ERs in patients with severe traumatic brain injury [20, 21]. In previous studies, the time to CT, time to definitive therapy (including thoracotomy or laparotomy), and time to TAE in trauma workflows in hybrid ERs were significantly reduced compared to those in non-hybrid ERs [19, 22]. A reduction in the time to CT and intervention is one of the greatest advantages of a hybrid ER. However, the clinical effects of hybrid ERs in trauma treatment have not been clearly defined. In particular, the safety of the new trauma workflow for performing CT during the primary survey has not been evaluated. Therefore, this study was necessary to clarify the safety of hybrid ERs in trauma management. In this study, we demonstrated that trauma treatment in a hybrid ER is as safe as conventional treatment performed in a non-hybrid ER. This result suggests that it may be reasonable to use the hybrid ER system actively in patients with severe trauma.

In our trauma protocol for hybrid ERs, CT was performed instead of FAST and radiography of the chest and pelvis. Trauma surveys have emphasized that this three-point examination is simple and can be performed without patient transfer. In this regard, CT in hybrid ERs is similar to FAST and radiography of the chest and pelvis, as in conventional imaging surveys. Additionally, whole-body CT can reveal unknown findings in the primary survey, including intracranial bleeding, mediastinal hematoma, and retroperitoneal hematoma around the aorta or the kidney. This provides advantages over conventional methods; if this information is available during the primary survey, it can guide the appropriate management strategy. Moreover, patients can immediately undergo resuscitative surgery, including hemostasis, without transfer. Our results revealed that the time from arrival to CT and resuscitative interventions was significantly reduced with hybrid ER use compared to non-hybrid ER use. This may benefit patients requiring the shortest possible time from hemostasis to surgery or IVR. Moreover, if CT in the hybrid ER reveals an intracranial hematoma with concurrent massive abdominal bleeding, simultaneous craniotomy and resuscitative laparotomy can be performed in the emergency department without patient transfer. The hybrid ER has another advantage—two or more surgeries can be performed simultaneously in the ER.

In a previous study, trauma workflow using the hybrid ER system decreased the mortality of patients with severe trauma [19]. This is the first clinical effect of hybrid ER systems. However, our results showed that there was no difference in the survival of patients between the hybrid ER and non-hybrid ER groups. We also assessed the survival benefit (survival and unexpected survival rate) at < 50, 50–64, 65–79, and ≥ 80 years of age between the two groups. However, there were no significant differences in survival and unexpected survival rates between the non-hybrid ER and hybrid ER groups in the four age groups. Thus, it may be difficult to improve survival using a hybrid ER as the survival rate in the conventional group (non-hybrid ER) was high. A previous study has indicated that more severely injured patients can survive in a hybrid ER [23]. This study showed that the TRISS Ps of survival patients treated in the hybrid ER was lower than that of survival patients treated in the non-hybrid ER. This suggests that hybrid ERs may contribute to survival in patients with more severe trauma and a high ISS (e.g., ISS ≥ 36).

We also focused on the amount of blood transfused because massive transfusion plays an important role in resuscitation during trauma treatment. Massive transfusion is a concept that forms the basis of DCR as evidence for the efficacy of DCR has been reported extensively in the literature [15, 16]. To determine the effect of blood transfusion in the hybrid ER, we compared the amount of blood transfused between the study groups. Our results revealed that the hybrid ER significantly reduced the amount of blood transfused compared to the non-hybrid ER. To our knowledge, this is the first report on the effectiveness of blood transfusion in a hybrid ER. A hybrid ER can provide accurate injury information and reduce the time required for hemostasis. Thus, the hybrid ER may be help in reducing the requirement of blood transfusions. One hypothesis is that faster hemostasis using a hybrid ER may reduce blood transfusion during trauma resuscitation. However, although we examined the correlation between the amount of blood transfused and time to CT or interventions, we could not confirm the correlation. Therefore, further investigation is needed to understand the factors contributing to lower transfusion rates in the hybrid ER.

Our study has some important limitations. First, our study was a retrospective study conducted at a single institution. Since there may be several biases in this study, the study results should be verified in a prospective, multicenter study. Second, our study could not identify whether trauma resuscitation was a suitable criterion for admission to a hybrid ER. Although patients with severe trauma and ISS ≥ 16 were analyzed in our study, suitable clinical criteria for a hybrid ER should be assessed in the future. Third, the prehospital information was not considered in this study because our database does not have this information. Time from injury to treatment and prehospital protocol may affect our results. Although we believe that the impact of prehospital information is little, it should be assessed in the future. Fourth, a significant barrier to the installation of a hybrid ER is the cost. We did not assess the costs and benefits of using a hybrid ER for trauma treatment. A hybrid ER is typically used as a CT room in an ER. Even if surgery is not performed for all patients, it may be possible to pay for a hybrid ER by performing CT a certain number of times in a day. In Japan, a dual room-type hybrid ER has recently been developed, considering its cost merit [24]. The cost-effectiveness of the hybrid ER should be estimated accordingly. Finally, the radiation exposure dose for whole-body CT in both groups was not assessed. Radiation exposure should be reduced for medical examination; therefore, an accurate dose of radiation in both groups should be assessed in the future.

## Conclusions

The new trauma workflow using whole-body CT in a hybrid ER is as safe as the conventional trauma protocol in a non-hybrid ER. The use of this protocol in the hybrid ER does not adversely affect patient prognosis, including survival and PTD. Moreover, a hybrid ER can reduce the amount of blood transfused during resuscitation, although the hybrid ER does not contribute to improved survival in patients with severe trauma and ISS ≥ 16. Importantly, the hybrid ER, which does not require patient transfer, has the advantage of reduced time to interventions. If the hybrid ER is used appropriately, early hemostasis can be achieved, and patients with severe trauma can benefit from reduced transfusion accordingly.

## Data Availability

The datasets used and/or analyzed during the current study are available from the corresponding author upon reasonable request.

## Abbreviations

*ER*: Emergency room
*ATLS*: Advanced trauma life support
FAST: Focused assessment with sonography for trauma
CT: Computed tomography
IVR: Interventional radiology
TAE: Transcatheter arterial embolization
DCR: Damage control resuscitation
JATEC: Japan Advanced Trauma Evaluation and Care
mFACT: Modified focused assessment with CT for trauma
AIS: Abbreviated Injury Scale
ISS: Injury Severity Score
RTS: Revised Trauma Score
Ps: Probability of survival
TRISS: Trauma and Injury Severity Score
CPA-OA: Cardiac pulmonary arrest on arrival
GCS: Glasgow Coma Scale
ACDiT: Adapted Clavien-Dindo in trauma
RBC: Red blood cell
FFP: Fresh frozen plasma
IQR: Interquartile range
CI: Confidence interval
